# Survey data on users perception of flexibility of spaces in selected cultural center in southwest Nigeria

**DOI:** 10.1016/j.dib.2018.06.099

**Published:** 2018-07-02

**Authors:** Adedapo Oluwatayo, Adedotun O. Akinola, Tosin Babalola, Hilary I. Okagbue, Samuel Olademehin, Segun Eyiaro, Samuel Oludara, Ometaghogho Johnson, Oluwasina Famurewa, Obiora Obi, Adebambo Adewakun, Ekara N. Ekara

**Affiliations:** a Department of Architecture, Covenant University, Ota, Nigeria; b Department of Mathematics, Covenant University, Ota, Nigeria

**Keywords:** Flexibility, Architecture, Design, Space use, Cultural center

## Abstract

Architects that specialize in designing cultural centers have often been accused of providing spaces that become obsolete in the coming years. This is because as technology and time changes, requirements also change, necessitating new arrangement of spaces. Very few of the spaces provided in cultural centers can however be adapted to other uses. This has affected the sustainability of those spaces. These data present the perceptions of users on the need for, and the features that enhance flexibility in cultural centers. The data were obtained from a questionnaire survey of users of the three (3) cultural centers in Nigeria. The survey was conducted between October and November 2017. The data may facilitate the evidence-based approaches to facilitate improved built environment and will be useful to built environment professionals, policy makers and design researchers.

**Specifications Table**

**Table:** 

Subject area	*Architecture*
More specific subject area	*Flexible and adaptable spaces in architectural designs*
Type of data	*Tables and Figures*
How data was acquired	*Field Survey through questionnaire*
Data format	*Raw and analyzed*
Experimental factors	*Both purposive and random sampling techniques were used in the selection of respondents in the survey*
Experimental features	*The data were obtained from the users of the three (3) cultural Centers: June 12 Cultural Centre, Ogun State, National Theatre, Lagos State and Oyo Cultural Centre, Oyo State. Data were analyzed using descriptive statistics.*
Data source location	*Ogun, Oyo and Lagos States, Nigeria*
Data accessibility	*All collected data are in this data article*

**Value of the data**

• Descriptive statistics were used in the presentation of the dataset which if analyzed will help reveal the factors that affects the use of cultural centers and features that can enhance flexibility in the use of cultural center spaces.
• The data could be used in development of design standards in the design of cultural buildings.
• The data could be used as bases for comparison of flexibility of spaces in cultural center in different countries.
• The data can directly help building design professionals take appropriate decisions in the design of cultural center in Nigeria.
• The dataset can be useful to the government and private developers as a guide in addressing the issue of flexibility in design of other cultural center and similar buildings taking into consideration the users׳ perception.
• The work is a major improvement over [1], [2], [3], [4], [5], [6], [7], [8], [9].

## 1 Data

**Fig. 1:**
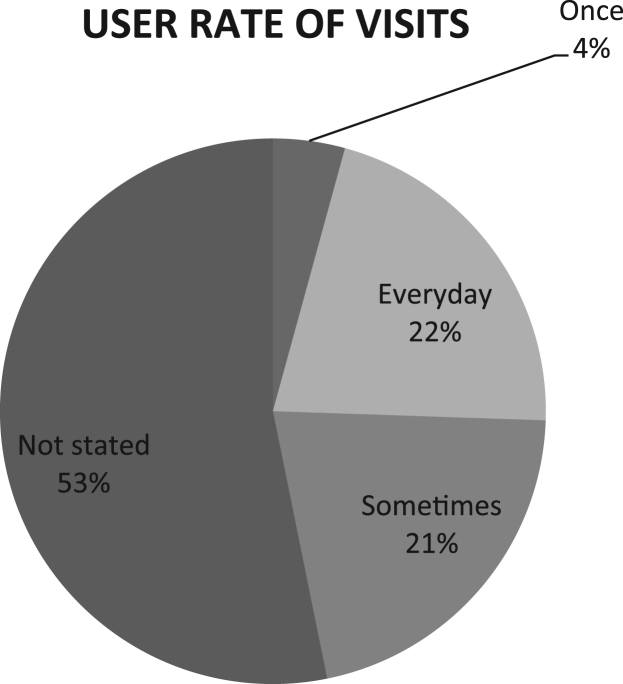
Number of times users visited the center.

**Fig. 2:**
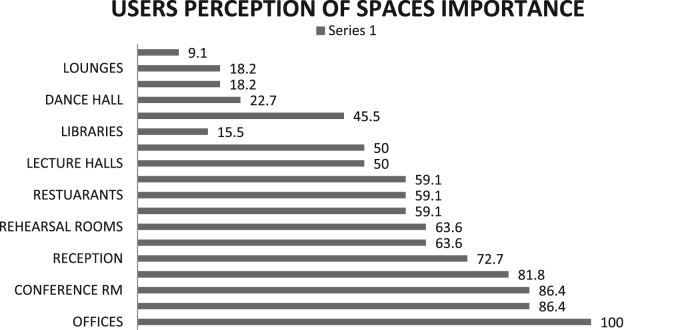
Users׳ perception of space allocation in cultural center by importance.

**Fig. 3:**
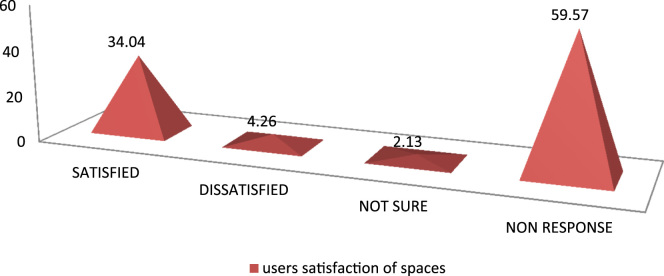
Users satisfaction of spaces.

The data were drawn out of survey of three (3) cultural centers in South West, Nigeria. The data instrument for the study is the questionnaire containing both open and close-ended questions with each variable measured on the Likert-like five-point scale. Forty-six out of fifty questionnaires administered for the users of the selected cultural center were returned. The data were collected between October and November 2017. The data collected were analyzed using Statistical Package for Social Science (SPSS). Frequencies and mean score rankings were carried out. Table 1 shows users purpose of visit, Fig. 1 reveals number of times users visited the Center, Fig. 2 describes the respondents׳ perception of space allocation in cultural center, while Fig. 3 shows users satisfaction of spaces. Also, Table 2 shows details of the reasons why flexibility is important in the design of cultural center and Table 3 shows respondents׳ perception of features that enhances flexibility in the design of cultural center.

**Table 1:** Respondents purposes of visit in percentage.

**S/n**	Purpose of visit	Percentage
**1.**	Cultural events	25
**2.**	Work and business	25
**3.**	Leisure	13.7
**4.**	Excursion	9.1
**5.**	Religious	4.5
**6.**	No response	22.7

**Table 2:** Reasons why flexibility is important in the design of cultural center.

		**Percent of cases**
For which reason is flexibility important?	Change in function	89.1
	To achieve more space	60.9
	Change in technology	58.7
	Change in pattern of use	47.8
	Promoting user comfort	37.0
	Locating activities with close relationships together	19.6

**Table 3:** Respondents׳ perception of features that enhances flexibility in the design of cultural center.

	**Mean**	**Std. deviation**
Use of sliding panels and doors	4.1957	0.45312
Use of demountable partitions	4.1304	0.40048
Use of compatible furniture	4.1304	0.49927
Appropriate technology	4.1087	0.52613
Lightweight internal construction	4.0870	0.58977
Use of movable furniture	4.0870	0.55080
Use of movable partitions	4.0652	0.32675
Open floor plans	4.0435	0.55604
Use of stackable furniture	4.0222	0.49949
Appropriate Lighting	4.0217	0.74503
Use of space-saving furniture	4.0217	0.44667
Proper acoustic treatment	4.0000	0.66667
Use of multi-functional furniture	3.9783	0.57693
Use of reconfigurable furniture	3.9783	0.44667
Proper electrical planning e.g. Raised floors	3.9783	0.61424
Frame construction	3.9565	0.55604
Use of soft space dividers and curtain walls	3.9348	0.53342
Use of operable walls	3.8913	0.60473
Minimal use of load-bearing supports	3.8478	0.59507
Providing spaces for future expansion	3.8043	0.65386
Locating activities with close relationships together	3.5652	0.77895
Open-ended corridors	3.5435	0.72131
Façade openings and screens	3.5000	1.00554
Multi-functional floor design	3.4783	0.75245
Multifunctional ceiling design	3.4565	0.88711
Grid design/concept	3.3333	0.85280
Use of retractable panels, roofs, floors, etc.	3.2826	0.88602
Use of responsive building elements	3.0000	0.81650

## 2 Experimental design, materials and methods

A survey of users of three (3) cultural centers was carried out in South West, Nigeria. The cultural centers investigated are June 12 cultural center, Ogun State, National Theatre, Lagos State and Oyo Cultural center, Oyo state. Studies [10], [11], [12], [13], [14], [15], [16], [17], [18], [19], [20] have adopted a similar approach in obtaining empirical data. The sources of data used in this research were primary. Fifty (50) questionnaires containing close and open-ended questions were administered to random sampled users of the three (3) cultural centers in southwest Nigeria. A response rate of 92% was recorded. Data obtained were collected between October and November, 2017. Data collected were analyzed using Statistical Package for Social Science (SPSS). The data on respondents’ profiles were analyzed using multiple responses (frequencies).

A sample of the questionnaire used is presented as Supplementary data A. A careful examination of the questionnaire reveals that it has three main sections. Section A was used to extract data on the socio-economic characteristics of the respondents, and their levels of satisfaction with the facilities provided in the cultural center. The data were extracted using 5-Likert type scale, where 1 represents “Very Dissatisfied”; 2 represents “Dissatisfied”; 3 represents “Undecided”; 4 is for “Satisfied”; and 5 represents “Very Satisfied”. Section B had questions on the extent of importance of spaces provided in cultural center in a scale of 1 to 5, where 1 represented “Totally Not Important”; and 5 “Very Important’. The last part of the questionnaire -Section C-, was used to gather data on the extent to which some features can enhance flexibility in the use of cultural center on a scale where 1=” Totally Not”; and 5 = “Large Extent”. Further analysis using different statistical tools can be done using the raw data presented as Supplementary data B.
