# Supplementary material for: Survey data on users perception of flexibility of spaces in selected cultural center in southwest Nigeria
Source: Data Brief. 2018 Jul 2;19:1888–93. doi: 10.1016/j.dib.2018.06.099 (PMC6141421; doi:10.1016/j.dib.2018.06.099)
Supplement: Supplementary file 1 — Supplementary material [file mmc1.pdf]

# COVENANT UNIVERSITY
## COLLEGE OF SCIENCE AND TECHNOLOGY
## DEPARTMENT OF ARCHITECTURE

CANAANLAND, KM 10, IDIROKO ROAD
P.M.B 1023, OTA, OGUN STATE, NIGERIA
www.covenantuniversity.edu.ng


EXTERNAL MEMO

**To:** Editor, Data in Brief
**From:** Corresponding Author
**Date:** 1st June, 2018
**Subject:** **Conflict of Interest**

I thereby declare the absence of any conflict of interest among the authors.

The authors have read the final draft and unanimously agreed that the paper be sent for review.

The source of funding and data have been acknowledged.


**Adedotun O. Akinola**
Department of Architecture, Covenant University, Nigeria
