# Supplementary material for: Survey data on users perception of flexibility of spaces in selected cultural center in southwest Nigeria
Source: Data Brief. 2018 Jul 2;19:1888–93. doi: 10.1016/j.dib.2018.06.099 (PMC6141421; doi:10.1016/j.dib.2018.06.099)
Supplement: Supplementary file 2 — Supplementary material [file mmc2.docx]

**QUESTIONNAIRE**

**OYO STATE ARTS AND CULTURE CENTRE: Achieving Flexibility in design**

Dear respondent,

This questionnaire is to find out on the design requirements of arts and culture centres, features that will be considered flexible in the design of arts and culture centres, challenges that have or could be encountered in the design of arts and culture centres and the impact flexibility features/strategies =have on the use of arts and culture centres. I promise to keep the information provided anonymous and only collated results will be publicized. Thank you for your co-operation.

**Olademehin Samuel O**.

Covenant University

**PART 1: PERSONAL PROFILE**

Please tick appropriate answers in spaces provided.

1. Marital Status: Married { } Widowed { } Separated { } Divorced { }

Single { } Prefer not to say { }

1. Age : Less than 21{ } 21-40 { } 41-60 { } 61 & above { }
2. Sex: Male { } Female { }
3. Highest Level of Education: Primary { } Secondary { } Bachelor’s Degree { } Master’s Degree { } Doctorate Degree { }
4. Job Status : Unemployed { } Self-employed { } Civil servant { } Private Organisation { } Student { }
5. Ethnicity : Yoruba{ } Ibo { } Hausa { } others { } Please Specify ________________

**PART 2: GENERAL QUESTIONS**

Please tick appropriate answers in spaces provided.

1. Have you ever visited a cultural centre? YES { } NO { }
2. For what purpose do you visit the centre? Tick as many as apply to you.

Cultural Event(S) { } Work & Business { } Excursion { }

Leisure { } Religious Activity { }

1. How often do you visit the cultural centre?

EVERYDAY { } SOMETIMES { } FIRST TIME { }

1. If No, or If not often, what are the Likely reasons? Tick as many as possible.

None known to me Location is far from me I don’t really have time

No attractive one Poor circulation Size of spaces are too small

It is hardly used Poor space planning Poor state of the facility

Spaces cannot be used for other activities Spaces provided are not comfortable

1. What facilities exist in the cultural centre? Please tick in front of as many as are there.

| Theatres / Auditoriums |  | **Museum** |  |
| --- | --- | --- | --- |
| Offices |  | **Library** |  |
| Exhibition Halls / Gallery |  | **Lecture Halls** |  |
| Conference Rooms |  | **Rehearsal Rooms** |  |
| Control / Technical Room |  | **Retail Shops** |  |
| Outdoor Amphitheatre |  | **Training Rooms** |  |
| Cafeteria/Restaurants |  | **Storage Rooms** |  |
| Tourist Info. Room |  | **Music Studio** |  |
| Kitchen |  | **Lounges** |  |
| Research Room |  | **Service Area** |  |
| Reception Area |  | **Dance Hall** |  |

1. How satisfied were you with the facilities provided when you visited the centre?

Very Satisfied { } Satisfied { } Undecided { } Disatisfied { }

Very Disatisfied{ }

1. To what extent are the following spaces important to cultural centres functioning? Tick as many as possible.

| **SPACES** | **Totally Not**  **Important** | **Not really**  **Important** | **Undecided** | **Important** | **Very Important** |
| --- | --- | --- | --- | --- | --- |
| **Theatres/Auditoriums** |  |  |  |  |  |
| **Offices** |  |  |  |  |  |
| **Museums** |  |  |  |  |  |
| **Library** |  |  |  |  |  |
| **Exhibition Halls** |  |  |  |  |  |
| **Conference Rooms** |  |  |  |  |  |
| **Lecture Halls** |  |  |  |  |  |
| **Cafeteria/Restaurants** |  |  |  |  |  |
| **Control Room** |  |  |  |  |  |
| **Retail Shops** |  |  |  |  |  |
| **Amphitheatres** |  |  |  |  |  |
| **Training rooms** |  |  |  |  |  |
| **Storage Room** |  |  |  |  |  |
| **Lounges** |  |  |  |  |  |
| **Rehearsal rooms** |  |  |  |  |  |
| **Storage rooms** |  |  |  |  |  |
| **Tourist Info. Room** |  |  |  |  |  |
| **Music Studio** |  |  |  |  |  |
| **Kitchen** |  |  |  |  |  |
| **Service Area** |  |  |  |  |  |
| **Dance hall** |  |  |  |  |  |

**PART 3: FEATURES THAT ENHANCE FLEXIBILE SPACES**

1. For which of the following reasons do you think flexibility in design is necessary? Tick as many as possible.

Change in pattern of use Change in Function Change in technology

Locating activities with close relationship together To achieve more space

Promoting user comfort

1. To what extent do you think the following features enhance flexibility in the use of cultural centres?

| **FEATURES** | **Totally Not** | **Not really** | **Undecided** | **Reasonable Extent** | **Large Extent** |
| --- | --- | --- | --- | --- | --- |
| Use of movable partitions |  |  |  |  |  |
| Frame construction |  |  |  |  |  |
| Use of demountable partitions |  |  |  |  |  |
| Appropriate Lighting |  |  |  |  |  |
| Proper electrical planning e.g. Raised floors |  |  |  |  |  |
| Use of reconfigurable furniture |  |  |  |  |  |
| Use of stackable furniture |  |  |  |  |  |
| Use of soft space dividers and curtain walls |  |  |  |  |  |
| Use of space-saving furniture |  |  |  |  |  |
| Use of retractable panels, roofs, floors, etc. |  |  |  |  |  |
| Multi-functional floor design |  |  |  |  |  |
| Use of responsive building elements |  |  |  |  |  |
| Minimal use of load-bearing supports |  |  |  |  |  |
| Lightweight internal construction |  |  |  |  |  |
| Use of operable walls |  |  |  |  |  |
| Proper acoustic treatment |  |  |  |  |  |
| Appropriate technology |  |  |  |  |  |
| Use of movable furniture |  |  |  |  |  |
| Use of compatible furniture |  |  |  |  |  |
| Use of sliding panels and doors |  |  |  |  |  |
| Use of multi-functional furniture |  |  |  |  |  |
| Façade openings and screens |  |  |  |  |  |
| Multifunctional ceiling design |  |  |  |  |  |
| Open floor plans |  |  |  |  |  |
| Open-ended corridors |  |  |  |  |  |
| Locating activities with close relationships together |  |  |  |  |  |
| Providing spaces for future expansion |  |  |  |  |  |
| Grid design/concept |  |  |  |  |  |

1. To what extent do you think the following constitute challenges in the design of cultural centres?

| **FEATURES** | **Large Extent** | **Reasonable Extent** | **Undecided** | **Not really** | **Totally Not** |
| --- | --- | --- | --- | --- | --- |
| Accessibility |  |  |  |  |  |
| Size of the facility |  |  |  |  |  |
| Provision of supporting facilities |  |  |  |  |  |
| Acoustics |  |  |  |  |  |
| Adaptability |  |  |  |  |  |
| Circulation |  |  |  |  |  |
| Expansion of facility |  |  |  |  |  |
| Location |  |  |  |  |  |
| Quality of the facility in terms of spaces provided |  |  |  |  |  |
| Use of proper planning standard |  |  |  |  |  |
| Lighting |  |  |  |  |  |
| Space programming |  |  |  |  |  |

Thank you so much for your time. GOD BLESS YOU!
